# Temporal patterns and adverse pregnancy outcomes of hypertensive disorders of pregnancy and gestational diabetes mellitus in Hebei Province, China, 2017–2023: a multicenter retrospective cohort study

**DOI:** 10.3389/fpubh.2026.1885150

**Published:** 2026-10-22

**Authors:** Li Liu, Suxin Liu, Liyan Du, Xiaoqian Yin, Yan Huo

**Affiliations:** 1 Department of Obstetrics and Gynecology, Hebei General Hospital, Shijiazhuang, China; 2 Department of Information Management, Hebei Center for Women and Children’s Health, Shijiazhuang, China

**Keywords:** adverse pregnancy outcomes, China, epidemiology, gestational diabetes mellitus, hypertensive disorders of pregnancy, preterm birth, retrospective cohort

## Abstract

**Background:**

Hypertensive disorders of pregnancy (HDP) and gestational diabetes mellitus (GDM) are common complications with overlapping metabolic, vascular, and inflammatory pathways. GDM is associated with fetal overgrowth but may also coexist with fetal growth restriction, particularly when placental vascular dysfunction is present. Regional evidence on recorded temporal patterns and joint maternal-neonatal outcomes remains limited in northern China.

**Methods:**

We analyzed 264,278 eligible singleton deliveries at ≥28 gestational weeks recorded in the Hebei Obstetric Critical Illness Monitoring System across 31 hospitals from 2017 to 2023. Pregnancies were classified as HDP alone, GDM alone, coexisting HDP and GDM, or unaffected. Multivariable logistic regression adjusted for maternal age, marital status, education, hospital level, gravidity, parity, prenatal examination frequency, and previous cesarean section. Sensitivity analyses examined center composition and hospital-specific random intercepts. Associations were interpreted as non-causal because important metabolic and behavioral covariates were unavailable.

**Results:**

Of eligible deliveries, 11,708 (4.43%) had HDP alone, 18,600 (7.04%) had GDM alone, 2,138 (0.81%) had coexisting HDP-GDM, and 231,832 (87.72%) were unaffected. The recorded prevalence of HDP alone rose from 3.42% in 2017 to 7.60% in 2023. GDM alone and coexisting HDP-GDM peaked in 2021 (13.21 and 1.39%) and then fell abruptly in 2022–2023. HDP alone showed the strongest associations with placental abruption, preterm delivery, small-for-gestational-age birth, and neonatal asphyxia; GDM alone was associated with macrosomia and premature rupture of membranes; the combined group had a mixed risk profile. Estimates for stillbirth and puerperal infection in the combined group were imprecise because only one and two events, respectively, occurred.

**Conclusion:**

HDP, GDM, and their coexistence were associated with distinct but overlapping outcome profiles. The abrupt post-2021 decline in recorded GDM persisted in center-composition sensitivity analyses but cannot be interpreted as a confirmed population-level decline because screening and ascertainment differences remain unresolved. The results support cautious, guideline-concordant integrated surveillance rather than study-derived monitoring thresholds.

## Introduction

Pregnancy requires profound metabolic and cardiovascular adaptations to support placental and fetal development, including increased insulin resistance, expanded plasma volume, altered lipid metabolism, and changes in vascular tone. Failure of these compensatory processes contributes to gestational metabolic and vascular disorders such as GDM and HDP (1, 2).

GDM and HDP are among the most common pregnancy complications and are associated with substantial short- and long-term maternal and neonatal morbidity. GDM is classically associated with macrosomia, cesarean delivery, birth trauma, neonatal hypoglycemia, and later maternal type 2 diabetes; however, fetal growth restriction or small-for-gestational-age birth may also occur when placental vascular dysfunction or preeclampsia coexists (1, 3–5). HDP is associated with placental insufficiency, fetal growth restriction, placental abruption, preterm birth, maternal organ dysfunction, and future cardiovascular disease. A meta-analysis also found that women with prior HDP have a substantially increased risk of developing chronic hypertension (2, 6, 7).

The coexistence of GDM and HDP is increasingly relevant as maternal age, overweight and obesity, assisted reproduction, and chronic metabolic disease increase. Li et al. reported that concomitant GDM and preeclampsia were associated with higher risks of preterm birth, cesarean delivery, and fetal growth restriction, while other large cohorts have shown that combined phenotypes do not produce uniformly additive risks across all outcomes (4, 5, 8, 9). Shared mechanisms may include insulin resistance, chronic inflammation, oxidative stress, endothelial dysfunction, and abnormal placentation (10, 11).

A large provincial monitoring system in Hebei provides an opportunity to describe recorded annual patterns and compare outcome profiles across HDP alone, GDM alone, and coexisting disease. However, surveillance estimates can be influenced by changing hospital participation, screening uptake, laboratory procedures, and diagnostic coding or reporting, particularly across a seven-year multicenter period (12, 13). This study therefore aimed to describe recorded annual patterns from 2017 to 2023 and estimate associations with maternal and neonatal outcomes. The analyses were intended to be associational rather than causal.

## Materials and methods

### Study design, setting, and reporting

This retrospective multicenter cohort study used routinely collected data from the Hebei Obstetric Critical Illness Monitoring System. The analysis included deliveries recorded by 31 hospitals in Hebei Province, China, between 1 January 2017 and 31 December 2023. The manuscript was revised in accordance with the STROBE principles for observational studies.

Hebei Province is located in northern China and includes plains, mountains, coastal areas, and urban–rural healthcare settings. These geographic and health-system differences increase the value of provincial surveillance but also make center-level participation, hospital grade, annual delivery volume, and diagnostic procedures essential for interpreting temporal patterns.

Supplementary Tables S1A,B describe the 31 participating hospitals, including location, hospital grade, participation period, mean annual eligible delivery volume, testing platform, sample handling, and quality control. The annual numbers of participating hospitals were 28, 30, 31, 28, 24, 16, and 14 from 2017 through 2023. The center roster lists 11 hospitals as participating throughout the study period; the reported stable-center sensitivity analysis included 10 hospitals.

### Participants

The source dataset contained 296,422 delivery records. Eligible records were singleton deliveries at ≥28 gestational weeks with complete core maternal and perinatal outcome information. The analysis included live births and the 135 stillbirths reported in Table 1; stillbirth was not a blanket exclusion criterion. Exclusions comprised gestational age <28 weeks, multiple gestation, fetal-condition exclusions, and incomplete or otherwise ineligible records. The final analytic sample comprised 264,278 deliveries. Figure 1 summarizes selection, with the scope of the original combined fetal-exclusion category requiring reconciliation.

**Table 1:** Maternal and neonatal outcomes by exposure group.

Outcome	HDP alone (*n* = 11,708)	GDM alone (*n* = 18,600)	HDP+GDM (*n* = 2,138)	Unaffected (*n* = 231,832)
Cesarean section	8,866 (75.73)	11,039 (59.35)	1,579 (73.85)	120,075 (51.79)
Placental abruption	166 (1.42)	58 (0.31)	17 (0.80)	89 (0.04)
Premature rupture of membranes	373 (3.19)	1,564 (8.41)	120 (5.61)	13,238 (5.71)
Puerperal infection	9 (0.08)	4 (0.02)	2 (0.09)	26 (0.01)
Postpartum hemorrhage	263 (2.25)	395 (2.12)	64 (2.99)	3,006 (1.30)
Anemia	4,966 (42.42)	7,397 (39.77)	962 (45.00)	71,952 (31.04)
Hypothyroidism	495 (4.23)	885 (4.76)	131 (6.13)	8,058 (3.48)
Term delivery (≥37 weeks)	9,031 (77.14)	17,336 (93.20)	1,743 (81.52)	221,100 (95.37)
Late preterm delivery (34–36 + 6 weeks)	1,652 (14.11)	954 (5.13)	282 (13.19)	8,262 (3.56)
Early preterm delivery (28–33 + 6 weeks)	1,025 (8.75)	310 (1.67)	113 (5.29)	2,470 (1.07)
Macrosomia	801 (6.84)	2,123 (11.41)	219 (10.24)	19,545 (8.43)
Small for gestational age	1,556 (13.29)	241 (1.30)	172 (8.04)	3,304 (1.43)
Neonatal asphyxia	247 (2.11)	104 (0.56)	36 (1.68)	1,243 (0.54)
Stillbirth	18 (0.15)	13 (0.07)	1 (0.05)	103 (0.04)

Values are *n* (%) using the displayed exposure-group denominators. SGA, small for gestational age. Stillbirth and puerperal infection in the HDP+GDM group were based on 1 and 2 events, respectively. Neonatal-asphyxia percentages are displayed per all eligible deliveries; the live-birth risk set for its adjusted model requires clarification (Q4).

**Figure 1:**
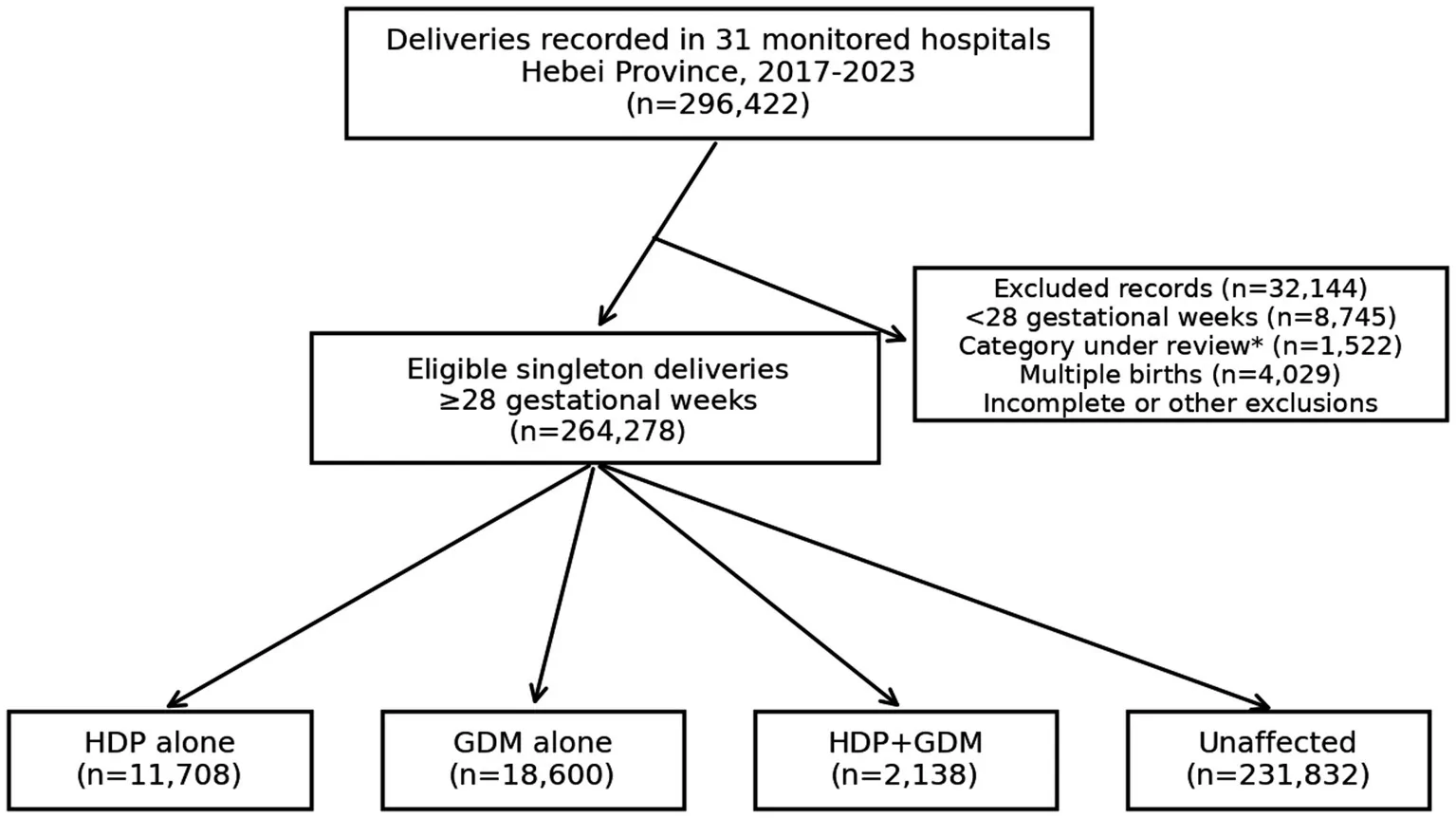
Flow of records from the source registry to the final analytic sample. The eligibility wording includes the 135 retained stillbirths. *The original 1,522-record fetal-death/malformation exclusion category requires reconciliation with these retained events (Q2); its count has not been reassigned.

### Exposure definitions

Pregnancies were categorized into four mutually exclusive groups: HDP alone, GDM alone, coexisting HDP and GDM, and pregnancies without either recorded condition.

GDM was defined using a 75-g oral glucose tolerance test performed at 24–28 gestational weeks. A diagnosis was assigned if any venous plasma glucose threshold was met or exceeded: fasting 5.1 mmol/L, 1-h 10.0 mmol/L, or 2-h 8.5 mmol/L (14, 15).

Random plasma glucose alone was not used to assign GDM in this analysis. Pregestational diabetes and overt diabetes first recognized in pregnancy were excluded from the four-group comparison. For an incomplete OGTT, GDM was assigned when at least one available OGTT value met the applicable diagnostic threshold and overt diabetes had been excluded; otherwise glycemic status was considered indeterminate and the record was excluded. An incomplete test without a diagnostic value was not treated as evidence of normal glucose tolerance.

HDP was analyzed as the recorded composite exposure. Clinical classifications distinguish gestational hypertension, preeclampsia, eclampsia, chronic hypertension in pregnancy, and chronic hypertension with superimposed preeclampsia. Hypertension is generally defined as systolic blood pressure ≥140 mmHg and/or diastolic blood pressure ≥90 mmHg on repeated measurements. Postpartum reassessment helps distinguish gestational from chronic hypertension; normalization within 12 weeks postpartum is not required to diagnose every HDP subtype during pregnancy (16–18). The available reporting does not support reliable subtype-specific estimates (Supplementary Table S3).

#### Laboratory testing and ascertainment

The participating centers used enzymatic hexokinase or glucose-oxidase methods on Roche, Beckman Coulter, Abbott, Siemens, Mindray, or Hitachi chemistry platforms. Reported procedures included venous plasma collection after an 8-12-h fast, centrifugation within 30–60 min, analysis within 2 h, daily internal quality control, and provincial or inter-laboratory external quality assessment. Center-specific systems and procedures are summarized in Supplementary Tables S1A,B. Annual OGTT eligibility and completion denominators were unavailable for evaluating screening coverage.

### Outcomes and covariates

Maternal outcomes included cesarean delivery, placental abruption, premature rupture of membranes, puerperal infection, postpartum hemorrhage, anemia, and hypothyroidism. Postpartum hemorrhage was defined as blood loss ≥500 mL after vaginal delivery or ≥1,000 mL after cesarean delivery within 24 h after birth.

Perinatal outcomes included gestational age at delivery, late preterm delivery (34–36 + 6 weeks), early preterm delivery (28–33 + 6 weeks), macrosomia (birth weight >4,000 g), small for gestational age (birth weight below the 10th percentile for gestational age), neonatal asphyxia, and stillbirth.

Covariates included maternal age, marital status, education, hospital level, gravidity, parity, prenatal examination frequency, and previous cesarean section. Important potential confounders were unavailable, including pre-pregnancy body mass index, gestational weight gain, smoking and alcohol use, assisted reproductive technology, family history of metabolic disease, medication exposure, treatment adherence, and dietary factors. Detailed pre-existing blood-pressure and glycemic histories were not available for adjustment. The exclusion of recorded pregestational or overt diabetes does not guarantee the absence of unrecognized pre-existing dysglycemia. Residual confounding was therefore expected.

### Statistical analysis

Continuous variables were summarized as mean ± standard deviation or median with interquartile range, depending on distribution. Categorical variables were summarized as number and percentage. Between-group differences were evaluated using analysis of variance, Kruskal-Wallis tests, chi-square tests, or Fisher exact tests as appropriate. Bonferroni correction was used for multiple pairwise comparisons.

Annual recorded prevalence was calculated as the number of cases in each exposure group divided by the number of eligible deliveries captured in that year. Temporal patterns were assessed descriptively; no causal or population-incidence interpretation was assigned to year-to-year changes.

Annual patterns were reassessed in the 10-hospital stable-center analysis set. Center-standardized annual estimates were obtained from a binomial mixed model with a hospital-specific random intercept and calendar-year indicators. These results are presented in Supplementary Table S1C and were used to assess the sensitivity of the recorded pattern to center composition, not to establish population incidence.

Multivariable logistic regression models used pregnancies without recorded HDP or GDM as the reference group and adjusted for maternal age, marital status, education, hospital level, gravidity, parity, prenatal examination frequency, and previous cesarean section. Odds ratios (ORs) and 95% confidence intervals (CIs) are reported. The models estimate adjusted associations and do not establish causality.

Outcome models were also fitted with a hospital-specific random intercept while retaining the original covariates. Original-model and hospital-adjusted ORs and 95% CIs are compared in Supplementary Table S1D. Hospital level and the hospital-specific random intercept represent different aspects of between-center variation; neither adjustment removes differences in diagnostic ascertainment.

Exploratory E-values were calculated for positive associations whose reported 95% CIs excluded 1. Adjusted ORs were converted to approximate risk ratios using the observed outcome proportion in the unaffected group, followed by the E-value calculation (19). The conversion used the unrounded reference-group event count and denominator. Because a marginal reference-group proportion was combined with an adjusted OR, these quantities are approximate sensitivity summaries rather than exact confounding bounds for a fitted conditional model. They do not account for misclassification, selection, or uncertainty in the reference-group proportion and were not used to support causal claims.

## Results

### Study population and recorded temporal patterns

Among 264,278 eligible singleton deliveries, 11,708 (4.43%) were classified as HDP alone, 18,600 (7.04%) as GDM alone, 2,138 (0.81%) as coexisting HDP-GDM, and 231,832 (87.72%) as unaffected by either recorded condition. The record-selection process and final exposure groups are shown in Figure 1. Annual counts and recorded prevalence are summarized in Table 2.

**Table 2:** Annual recorded counts and prevalence of HDP, GDM, and coexisting HDP-GDM.

Year	Eligible deliveries	HDP alone *n* (%)	GDM alone *n* (%)	HDP+GDM *n* (%)	Unaffected n
2017	64,629	2,208 (3.42)	3,084 (4.77)	566 (0.88)	58,771
2018	46,347	1,612 (3.48)	3,194 (6.89)	262 (0.57)	41,279
2019	46,121	1,649 (3.58)	4,237 (9.19)	386 (0.84)	39,849
2020	34,253	1,543 (4.50)	3,591 (10.48)	429 (1.25)	28,690
2021	27,645	1,300 (4.70)	3,652 (13.21)	383 (1.39)	22,310
2022	22,864	1,643 (7.19)	403 (1.76)	60 (0.26)	20,758
2023	22,419	1,703 (7.60)	439 (1.96)	52 (0.23)	20,225

The recorded prevalence of HDP alone rose from 3.42% in 2017 to 7.60% in 2023. GDM alone increased from 4.77% in 2017 to 13.21% in 2021, then declined to 1.76% in 2022 and 1.96% in 2023. Coexisting HDP-GDM increased to 1.39% in 2021 and then declined to 0.26% in 2022 and 0.23% in 2023. The decline between 2021 and 2022 was substantially larger than preceding annual changes and occurred while the number of deliveries captured by the registry also decreased. In the absence of center-year screening and reporting data, this discontinuity cannot be distinguished from changes in ascertainment, hospital participation, diagnostic coding, or case reporting. The patterns are visualized in Figure 2, and annual eligible delivery volume is shown in Figure 3.

**Figure 2:**
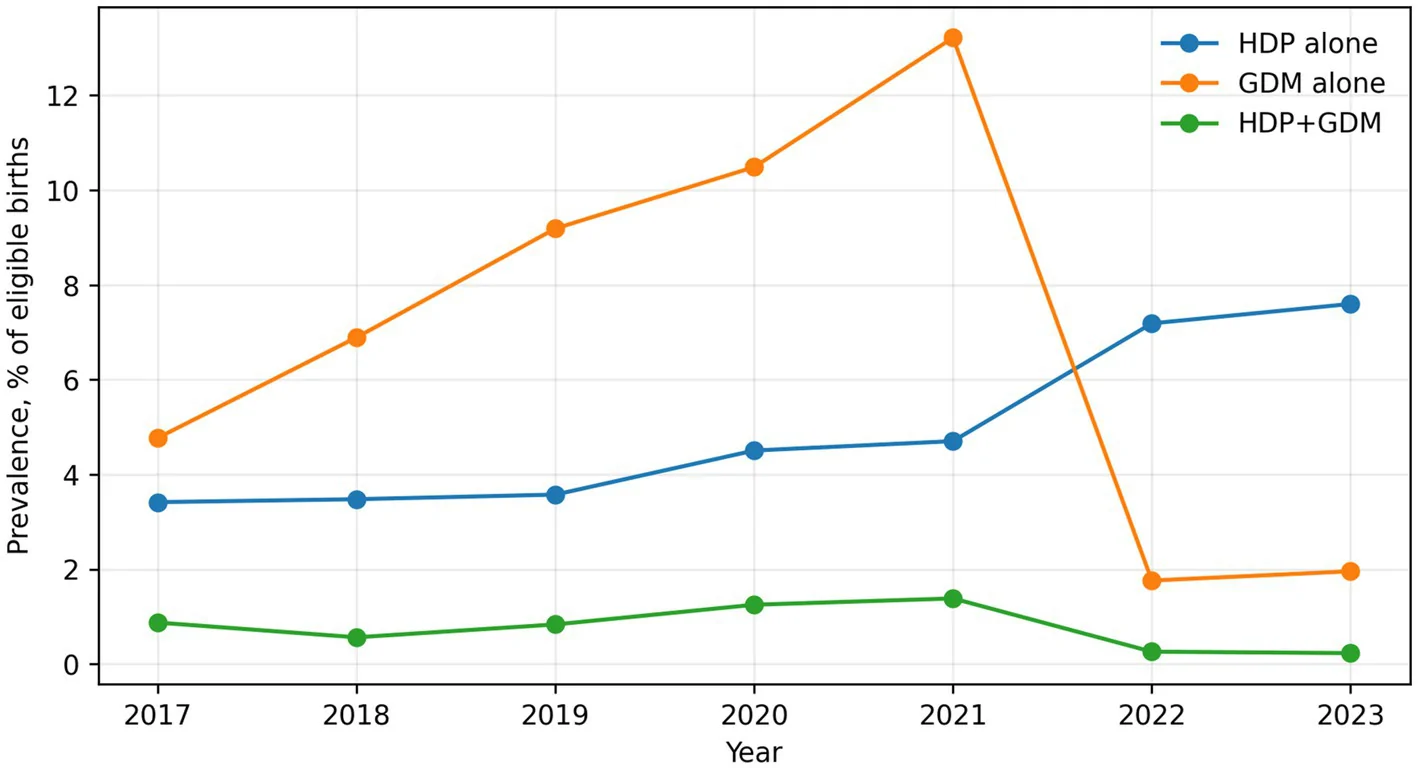
Recorded annual prevalence of HDP alone, GDM alone, and coexisting HDP-GDM per 100 eligible deliveries, 2017–2023. Values reproduce the supplied annual counts in Table 2; the 50-record annual-versus-overall exposure discrepancy remains flagged in Q1.

**Figure 3:**
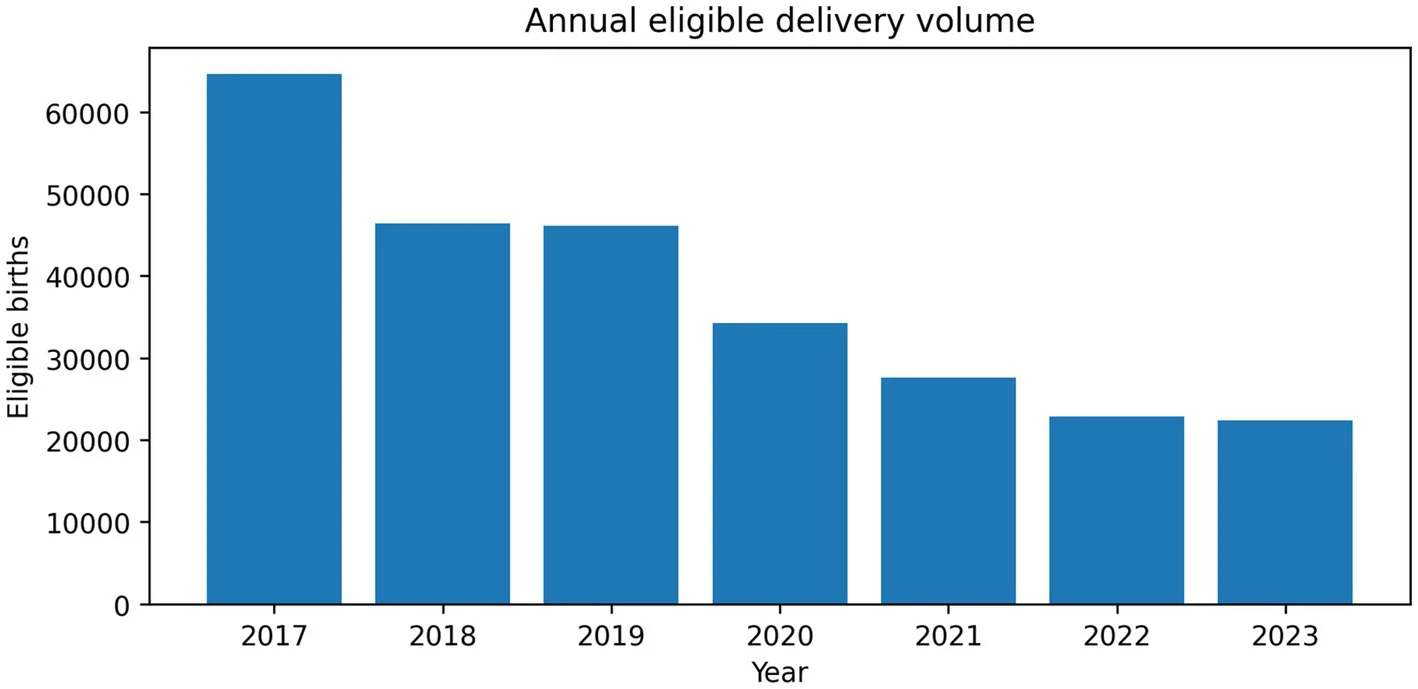
Annual number of eligible singleton deliveries captured in the monitoring system, 2017–2023.

### Center-level sensitivity analyses

In the 10-hospital stable-center analysis set, recorded GDM-alone prevalence was 4.90, 6.87, 9.08, 10.36, 12.58, 2.02, and 2.10% from 2017 through 2023; corresponding center-standardized estimates were 4.92, 6.84, 9.01, 10.29, 12.52, 2.10, and 2.18%. The post-2021 discontinuity persisted in these analyses, although the 2022–2023 values were higher than the all-center proportions. Hospital-random-intercept outcome estimates retained the direction of the original associations. Examples included OR 34.20 (95% CI, 24.80–47.16) for placental abruption with HDP alone, 1.39 (1.32–1.47) for macrosomia with GDM alone, and 5.18 (4.08–6.57) for early preterm delivery with coexisting HDP-GDM. The reported median hospital intraclass correlation coefficient was 0.047 (range, 0.018–0.109). The HDP-alone/hypothyroidism interval and the GDM-alone/stillbirth interval included 1 after hospital adjustment (Supplementary Tables S1C,D).

### Baseline characteristics

Maternal age differed across groups. Pregnancies complicated by HDP, GDM, or both had older maternal age distributions than unaffected pregnancies, and the coexisting HDP-GDM group had the highest proportion of women aged ≥40 years. Education, hospital level, gravidity, parity, prenatal examination frequency, and previous cesarean section also differed across groups. Detailed baseline characteristics are presented in Table 3.

**Table 3:** Key maternal demographic and obstetric characteristics by exposure group.

Characteristic	HDP alone (*n* = 11,708)	GDM alone (*n* = 18,600)	HDP+GDM (*n* = 2,138)	Unaffected (*n* = 231,832)	*p*-value
Age, years, mean ± SD	30.31 ± 4.48	30.75 ± 4.48	30.89 ± 5.05	29.47 ± 4.56	<0.001
Age ≥ 35 years	2,389 (20.4)	3,776 (20.3)	507 (23.7)	31,700 (13.7)	<0.001
Age ≥40 years	463 (4.0)	562 (3.0)	109 (5.1)	4,323 (1.9)	<0.001
University or above	5,294 (45.2)	9,992 (53.7)	967 (45.2)	104,097 (44.9)	<0.001
Municipal-level or higher hospital	7,715 (65.9)	13,935 (74.9)	1,639 (76.7)	114,861 (49.5)	<0.001
Gravidity ≥2	7,583 (64.8)	13,428 (72.2)	1,452 (67.9)	159,651 (68.9)	<0.001
Nulliparity	5,498 (47.0)	7,370 (39.6)	969 (45.3)	92,287 (39.8)	<0.001
Prenatal examinations ≥8	6,037 (51.6)	11,525 (62.0)	1,142 (53.4)	119,169 (51.4)	<0.001
Previous cesarean section ≥1	3,412 (29.1)	6,155 (33.1)	693 (32.4)	63,534 (27.4)	<0.001

Values are *n* (%) unless otherwise indicated.

### Maternal outcomes

Compared with unaffected pregnancies, all complication groups had higher crude rates of cesarean delivery, placental abruption, postpartum hemorrhage, anemia, and hypothyroidism. HDP alone and coexisting HDP-GDM were particularly associated with placental abruption, whereas GDM alone had the highest rate of premature rupture of membranes. Puerperal infection was rare in every group and occurred in only two pregnancies in the coexisting group, producing highly imprecise estimates. Crude maternal and perinatal outcome rates are summarized in Table 1.

After multivariable adjustment, HDP alone remained associated with cesarean delivery (OR 3.28, 95% CI 3.12–3.45), placental abruption (OR 37.44, 95% CI 28.13–49.84), postpartum hemorrhage (OR 1.72, 95% CI 1.51–1.97), anemia (OR 1.64, 95% CI 1.57–1.71), and hypothyroidism (OR 1.13, 95% CI 1.02–1.25). GDM alone was associated with cesarean delivery, placental abruption, premature rupture of membranes, postpartum hemorrhage, anemia, and hypothyroidism. Coexisting HDP-GDM was associated with cesarean delivery (OR 2.36, 95% CI 2.10–2.64), placental abruption (OR 18.90, 95% CI 10.61–33.65), postpartum hemorrhage (OR 2.32, 95% CI 1.78–3.03), anemia (OR 1.81, 95% CI 1.66–1.98), and hypothyroidism (OR 1.83, 95% CI 1.52–2.19). Full adjusted estimates are presented in Table 4 and Figures 4–6.

**Table 4:** Adjusted associations between pregnancy complications and outcomes.

Outcome	HDP alone OR (95% CI)	*P*	GDM alone OR (95% CI)	*P*	HDP+GDM OR (95% CI)	*P*
Cesarean section	3.280 (3.121–3.447)	<0.001	1.125 (1.083–1.169)	<0.001	2.358 (2.104–2.642)	<0.001
Placental abruption	37.441 (28.130–49.835)	<0.001	7.672 (5.334–11.033)	<0.001	18.898 (10.612–33.652)	<0.001
Premature rupture of membranes	0.468 (0.416–0.525)	<0.001	1.382 (1.303–1.466)	<0.001	0.907 (0.747–1.102)	0.327
Puerperal infection	6.548 (2.842–15.085)	<0.001	2.095 (0.712–6.162)	0.179	4.930 (0.660–36.816)	0.120
Postpartum hemorrhage	1.723 (1.506–1.972)	<0.001	1.698 (1.519–1.898)	<0.001	2.321 (1.779–3.028)	<0.001
Anaemia	1.639 (1.574–1.705)	<0.001	1.488 (1.441–1.537)	<0.001	1.810 (1.655–1.979)	<0.001
Hypothyroidism	1.133 (1.024–1.253)	0.016	1.307 (1.211–1.411)	<0.001	1.825 (1.520–2.193)	<0.001
Late preterm delivery (34–36 + 6 weeks)	9.990 (9.193–10.857)	<0.001	1.403 (1.304–1.510)	<0.001	3.988 (3.481–4.568)	<0.001
Early preterm delivery (28–33 + 6 weeks)	4.786 (4.501–5.088)	<0.001	1.540 (1.355–1.750)	<0.001	5.543 (4.509–6.814)	<0.001
Macrosomia	0.813 (0.752–0.879)	<0.001	1.413 (1.344–1.458)	<0.001	1.477 (1.288–1.694)	<0.001
Small for gestational age	10.268 (9.592–10.991)	<0.001	0.927 (0.809–1.062)	0.274	5.873 (4.967–6.944)	<0.001
Neonatal asphyxia	3.737 (3.219–4.340)	<0.001	1.053 (0.853–1.300)	0.629	2.981 (2.098–4.235)	<0.001
Stillbirth	3.119 (1.726–5.636)	<0.001	1.947 (1.056–3.589)	0.033	1.352 (0.188–9.742)	0.765

Reference group is pregnancies without recorded HDP or GDM. Adjusted for maternal age, marital status, education, hospital level, gravidity, parity, prenatal examination frequency, and previous cesarean section. These are associations rather than causal effects. ORs across exposure columns are not formal head-to-head comparisons; rare-event estimates should be interpreted cautiously. Hospital-random-intercept sensitivity estimates are reported in Supplementary Table S1D. Point estimates retained their direction, but the HDP-alone/hypothyroidism and GDM-alone/stillbirth CIs included 1 after hospital adjustment.

**Figure 4:**
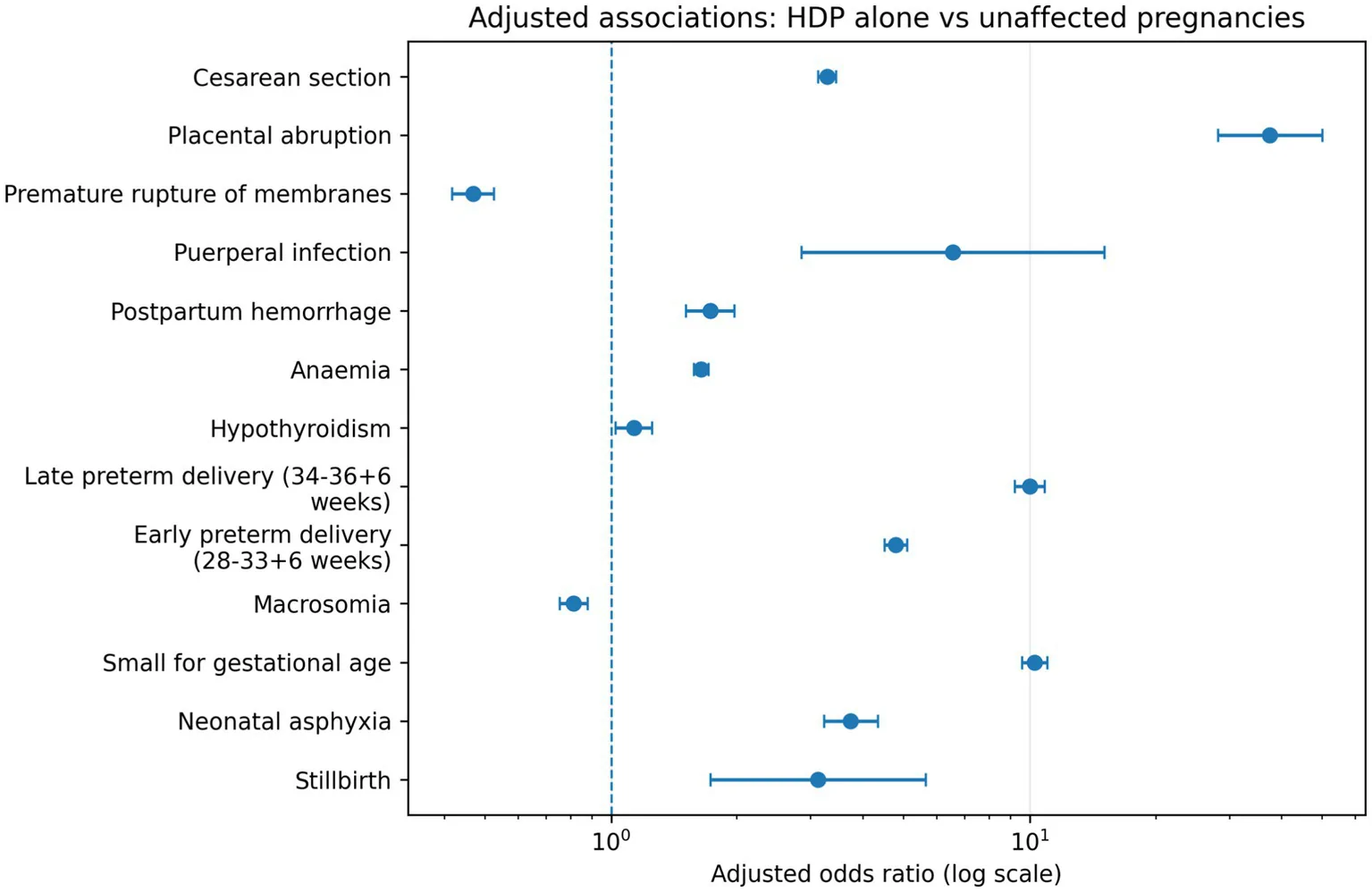
Adjusted odds ratios and 95% confidence intervals for HDP alone compared with unaffected pregnancies. The vertical reference line indicates OR = 1.

**Figure 5:**
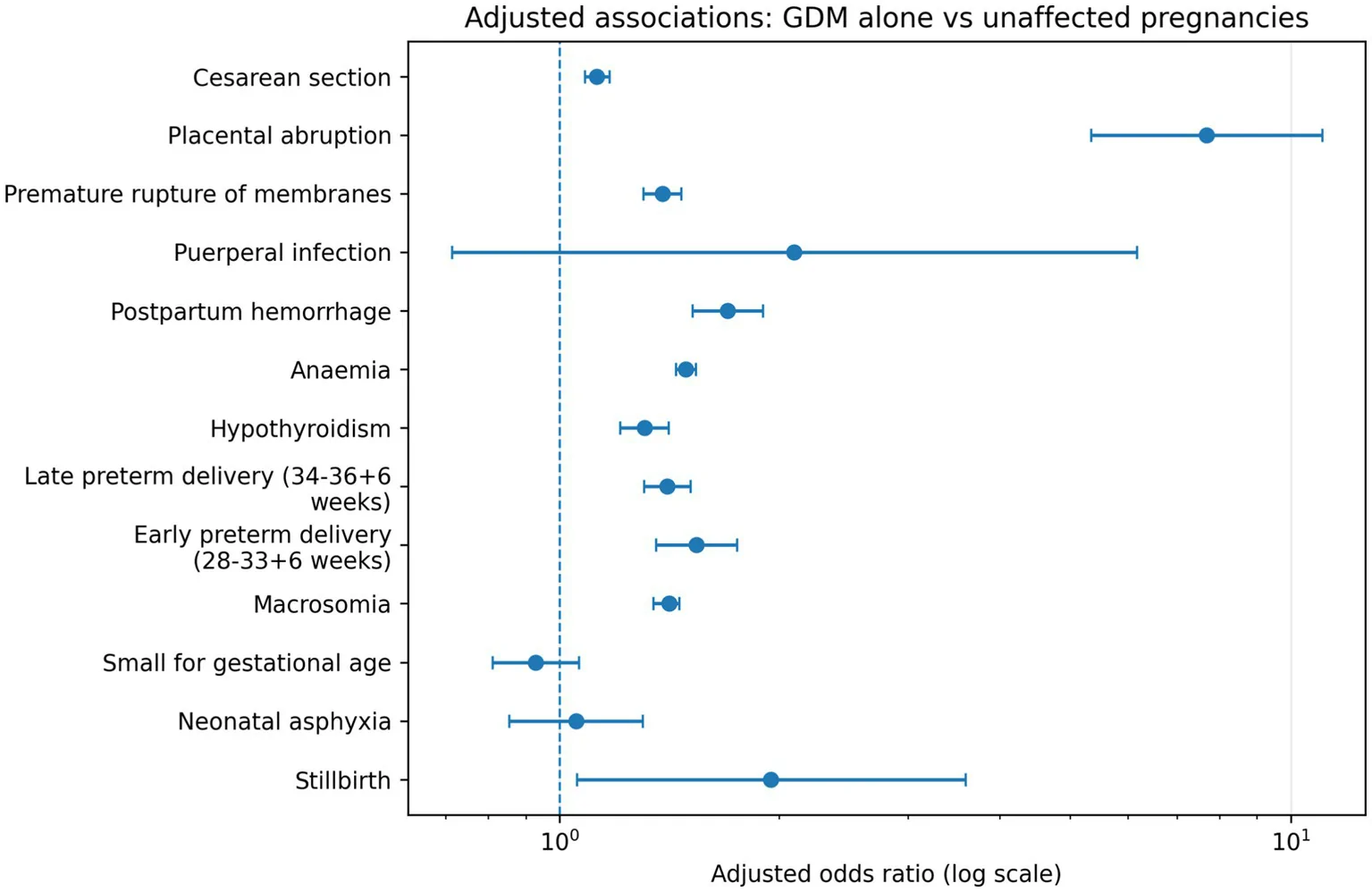
Adjusted odds ratios and 95% confidence intervals for GDM alone compared with unaffected pregnancies. The vertical reference line indicates OR = 1.

**Figure 6:**
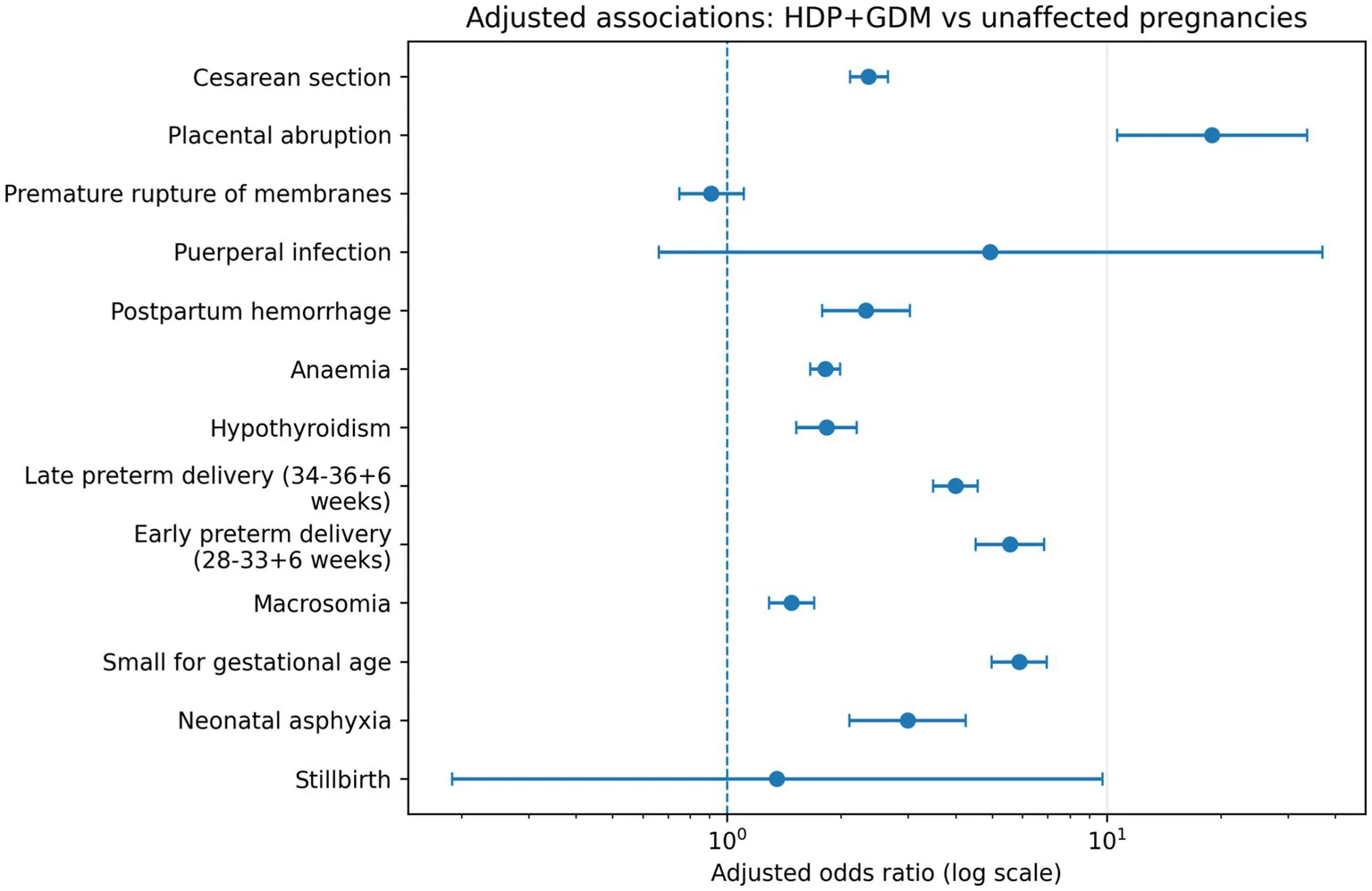
Adjusted odds ratios and 95% confidence intervals for coexisting HDP-GDM compared with unaffected pregnancies. The vertical reference line indicates OR = 1.

### Perinatal outcomes

HDP alone was associated with higher rates of both late and early preterm delivery, small-for-gestational-age birth, neonatal asphyxia, and stillbirth. GDM alone was associated with macrosomia and preterm delivery but not with small-for-gestational-age birth or neonatal asphyxia after adjustment. Coexisting HDP-GDM was associated with late preterm delivery (OR 3.99, 95% CI 3.48–4.57), early preterm delivery (OR 5.54, 95% CI 4.51–6.81), macrosomia (OR 1.48, 95% CI 1.29–1.69), small-for-gestational-age birth (OR 5.87, 95% CI 4.97–6.94), and neonatal asphyxia (OR 2.98, 95% CI 2.10–4.24). The stillbirth estimate for the coexisting group was not statistically significant (OR 1.35, 95% CI 0.19–9.74) and was based on one event; it therefore provides little information about the true stillbirth risk.

The combined HDP-GDM phenotype did not uniformly have higher point estimates than HDP alone. However, each exposure group was compared separately with the unaffected reference group; the differences between their ORs are not formal head-to-head comparisons and should not be interpreted as evidence that coexistence is protective. The placental abruption estimate in the combined group was based on 17 events and had a wide confidence interval.

### Exploratory sensitivity to unmeasured confounding

For coexisting HDP-GDM, the approximate E-values for the point estimate and lower confidence limit were 37.03 and 20.63 for placental abruption, 10.05 and 8.16 for early preterm delivery, 10.46 and 8.87 for small-for-gestational-age birth, 5.35 and 3.59 for neonatal asphyxia, and 2.19 and 1.83 for macrosomia. The sensitivity summaries therefore varied substantially by outcome. Supplementary Table S2 reports the calculations for all 31 positive associations whose reported 95% CIs excluded 1. These summaries do not demonstrate that confounding, selection, or ascertainment bias is absent.

## Discussion

### Principal findings

This multicenter retrospective cohort study describes recorded HDP, GDM, and coexisting HDP-GDM in Hebei from 2017 to 2023. Three findings are central. First, recorded HDP-alone prevalence increased, whereas GDM alone and coexisting HDP-GDM showed an abrupt discontinuity after 2021 that persisted in center-composition sensitivity analyses. Second, HDP alone had large adjusted associations with placental abruption, small-for-gestational-age birth, preterm delivery, and neonatal asphyxia; GDM alone was associated with premature rupture of membranes and macrosomia. Third, the combined phenotype showed an outcome-specific profile rather than uniformly higher point estimates. Several rare-outcome estimates were too imprecise to support firm conclusions.

#### Interpretation of the temporal discontinuity

The fall in recorded GDM-alone prevalence from 13.21% in 2021 to 1.76% in 2022, together with the parallel decline in coexisting HDP-GDM, cannot be confidently attributed to rapid population-level biological change. The decline persisted in the reported stable-center and center-standardized analyses, indicating that the modeled center-composition differences did not eliminate the discontinuity. These analyses do not rule out differences in antenatal attendance, screening completion, laboratory practice, diagnostic coding, or case reporting within hospitals. Annual OGTT eligibility and completion denominators and a comprehensive center-year audit of pandemic-related service changes were unavailable. The findings should therefore be described as registry-recorded patterns, not a confirmed public-health improvement. Recorded HDP patterns may likewise reflect both disease burden and ascertainment (12, 13, 20).

#### Clinical and biological interpretation of outcome-specific associations

The coexistence of metabolic and vascular disease need not produce uniformly larger associations than either recorded condition alone. HDP-related placental malperfusion, endothelial injury, and vasospasm may contribute to abruption and impaired fetal growth, whereas hyperglycemia and insulin resistance may contribute to fetal overgrowth (2, 10, 11). The lower placental-abruption OR for coexisting HDP-GDM than for HDP alone does not demonstrate a protective interaction: the models used the unaffected group as the reference and did not estimate a direct group contrast or interaction. Differences in HDP subtype and severity, delivery timing, surveillance or treatment, and residual confounding could influence the estimates; only 17 abruption events occurred in the combined group. Previous studies also report outcome-specific associations when GDM and preeclampsia or HDP coexist (4, 5, 9, 21, 22).

#### Residual confounding, rare outcomes, and negative findings

Residual confounding remains a major limitation. Pre-pregnancy obesity, gestational weight gain, assisted reproduction, smoking, medication exposure, family history, dietary factors, and incompletely characterized pre-existing vascular or metabolic disease may influence both exposure classification and pregnancy outcomes. Recorded pregestational and overt diabetes were excluded, but unrecognized pre-existing dysglycemia cannot be ruled out. Conversely, more intensive monitoring and earlier intervention among diagnosed women could attenuate observed associations. The approximate E-values provide a limited sensitivity summary under the specified conversion assumptions, not proof that the adjusted estimates are unbiased. Hospital adjustment also does not substitute for direct measurement of these covariates.

Null findings for rare outcomes should also be interpreted cautiously. The coexisting group contained only one stillbirth and two puerperal infections, resulting in very wide confidence intervals. The non-significant association with premature rupture of membranes in the combined group likewise does not establish absence of risk. Puerperal infection and stillbirth should be treated as exploratory secondary outcomes in this dataset rather than core indicators. Thyroid dysfunction was more frequent in the combined group, but this observational association alone is insufficient to define a universal serial-screening schedule (23).

#### Clinical implications

The study ORs should not be converted into unvalidated visit-frequency or testing thresholds. A more defensible implication is guideline-concordant integrated care: blood pressure and glycemic control should be reviewed together; fetal growth should be assessed serially according to clinical risk; umbilical-artery Doppler should be used when fetal growth restriction or placental dysfunction is suspected; and women with severe hypertension, abnormal growth or Doppler findings, or threatened early preterm delivery should be referred promptly to a tertiary center (14–18, 24). In primary hospitals, standardized screening and clear referral pathways are more appropriate than study-derived numerical cutoffs.

Postpartum follow-up is also important. Women with prior HDP have an increased long-term risk of chronic hypertension, and women with GDM have an increased risk of type 2 diabetes (6, 25, 26). The present findings therefore support coordinated postpartum blood-pressure and metabolic follow-up, while recognizing that the current registry did not evaluate long-term outcomes.

### Strengths and limitations

The strengths of this study include its large sample size, multicenter provincial coverage, and simultaneous assessment of HDP alone, GDM alone, and coexisting disease. The study evaluated a broad range of maternal and perinatal outcomes and provided event counts as well as adjusted estimates.

Several limitations require emphasis. First, the retrospective design and incomplete measurement of metabolic, behavioral, treatment, and family-history covariates preclude causal inference. Second, HDP was analyzed as a composite exposure, and subtype coding and postpartum blood-pressure ascertainment remain insufficiently characterized. Third, although center characteristics, testing procedures, and hospital-adjusted sensitivity estimates are now reported, annual OGTT eligibility and completion denominators, complete reagent-batch histories, and a comprehensive center-year screening and coding audit were unavailable. Fourth, the abrupt 2022–2023 GDM decline may reflect residual ascertainment or reporting differences despite center-composition adjustment. Fifth, some outcomes were very rare, and many associations were examined; isolated positive findings should not be treated as confirmatory. The reported models also did not include calendar year among the listed outcome-model covariates. Finally, approximate E-values based on a marginal reference-group risk do not remove confounding or other biases. The findings should be interpreted as registry-based associations rather than causal effects or definitive population trends.

## Conclusion

HDP, GDM, and their coexistence were associated with distinct but overlapping maternal and perinatal outcome profiles in the Hebei registry. HDP alone had large associations with placental and fetal-growth outcomes, whereas GDM alone was associated with macrosomia and premature rupture of membranes. The combined group did not have uniformly larger point estimates than HDP alone, but direct between-exposure contrasts and interactions were not tested. The abrupt post-2021 decline in recorded GDM persisted in center-composition sensitivity analyses and cannot be interpreted as a confirmed population decline given unresolved screening and ascertainment differences. The findings support cautious, guideline-concordant integrated care and prospective studies with standardized diagnostic procedures and comprehensive metabolic covariates.

## Data Availability

The raw data supporting the conclusions of this article will be made available by the authors, without undue reservation.
