# Supplementary material for: Temporal patterns and adverse pregnancy outcomes of hypertensive disorders of pregnancy and gestational diabetes mellitus in Hebei Province, China, 2017–2023: a multicenter retrospective cohort study
Source: Front Public Health. 2026 Oct 22;14:1885150. doi: 10.3389/fpubh.2026.1885150 (PMC13760690; doi:10.3389/fpubh.2026.1885150)
Supplement: Supplementary file 1 [file Data_Sheet_1.pdf]

Supplementary Material

Temporal patterns and adverse pregnancy outcomes of hypertensive disorders of pregnancy and gestational diabetes mellitus in Hebei Province, China, 2017-2023

Supplementary Table S1A. Characteristics, participation, delivery volume, and testing system of the 31 centers.

Center identifiers are masked consistently across the supplementary tables.

Center	Prefecture	Setting	Hospital grade	Participation	Mean annual eligible deliveries (range)	Analyzer/platform (manufacturer)	2021-2023 procedural/reporting note
C01	Shijiazhuang	Urban core	Grade IIIA	2017-2023	2,839 (2,296-4,259)	Roche cobas c702 (Roche Diagnostics)	No procedural change recorded in the center summary
C02	Shijiazhuang	Urban core	Grade IIIA	2017-2023	2,422 (1,565-3,843)	Beckman Coulter AU5800 (Beckman Coulter)	No procedural change recorded in the center summary
C03	Shijiazhuang	Urban peripheral	Grade IIIB	2017-2021	1,694 (1,172-2,359)	Hitachi 7180 (Hitachi High-Tech)	/
C04	Shijiazhuang	County/rural	Grade IIA	2017-2020	1,288 (901-1,738)	Hitachi 7180 (Hitachi High-Tech)	County-level scheduling variation; annual OGTT completion denominator unavailable
C05	Shijiazhuang	County/rural	Grade IIA	2018-2023	940 (754-1,265)	Beckman Coulter AU480 (Beckman Coulter)	Temporary reduction in scheduled OGTT capacity during parts of 2022; annual completion denominator not retained
C06	Tangshan	Urban core	Grade IIIA	2017-2023	2,213 (1,581-3,780)	Roche cobas c702 (Roche Diagnostics)	No procedural change recorded in the center summary
C07	Tangshan	Urban peripheral	Grade IIIA	2017-2021	1,648 (1,117-2,307)	Beckman Coulter AU5800 (Beckman Coulter)	/
C08	Tangshan	County/rural	Grade IIA	2017-2019	1,133 (908-1,465)	Roche cobas c501 (Roche Diagnostics)	County-level scheduling variation; annual OGTT completion denominator unavailable
C09	Baoding	Urban core	Grade IIIA	2017-2023	2,149 (1,568-3,746)	Siemens ADVIA Chemistry XPT (Siemens Healthineers)	No procedural change recorded in the center summary
C10	Baoding	Urban peripheral	Grade IIIA	2017-2022	1,633 (1,057-2,725)	Mindray BS-2000M (Mindray)	/
C11	Baoding	County/rural	Grade IIA	2017-2021	1,195 (903-1,573)	Beckman Coulter AU480 (Beckman Coulter)	No procedural change recorded in the center summary
C12	Baoding	County/rural	Grade IIA	2018-2023	813 (706-918)	Mindray BS-600M (Mindray)	Temporary reduction in scheduled OGTT capacity during parts of 2022; annual completion denominator not retained
C13	Handan	Urban core	Grade IIIA	2017-2023	2,024 (1,526-3,081)	Abbott ARCHITECT c16000 (Abbott)	No procedural change recorded in the center summary
C14	Handan	Urban peripheral	Grade IIIB	2017-2020	1,800 (1,304-2,434)	Mindray BS-800M (Mindray)	/
C15	Handan	County/rural	Grade IIA	2017-2021	1,054 (738-1,473)	Mindray BS-800M (Mindray)	No procedural change recorded in the center summary
C16	Cangzhou	Urban core	Grade IIIA	2017-2023	1,977 (1,448-3,133)	Roche cobas c702 (Roche Diagnostics)	No procedural change recorded in the center summary
C17	Cangzhou	County/rural	Grade IIA	2017-2019	1,177 (960-1,479)	Beckman Coulter AU480 (Beckman Coulter)	County-level scheduling variation; annual OGTT completion denominator unavailable
C18	Cangzhou	Urban peripheral	Grade IIIA	2019-2023	1,152 (955-1,350)	Abbott ARCHITECT c16000 (Abbott)	Temporary reduction in scheduled OGTT capacity during parts of 2022; annual completion denominator not retained
C19	Langfang	Urban core	Grade IIIA	2017-2023	1,758 (1,271-2,766)	Siemens ADVIA Chemistry XPT (Siemens Healthineers)	No procedural change recorded in the center summary

Center	Prefecture	Setting	Hospital grade	Participation	Mean annual eligible deliveries (range)	Analyzer/platform (manufacturer)	2021-2023 procedural/reporting note
C20	Langfang	County/rural	Grade IIA	2017-2021	1,202 (748-2,003)	Roche cobas c501 (Roche Diagnostics)	No procedural change recorded in the center summary
C21	Xingtai	Urban core	Grade IIIA	2017-2023	1,810 (1,321-2,938)	Roche cobas c702 (Roche Diagnostics)	No procedural change recorded in the center summary
C22	Xingtai	Urban peripheral	Grade IIIB	2017-2021	1,513 (1,082-2,356)	Beckman Coulter AU480 (Beckman Coulter)	/
C23	Xingtai	County/rural	Grade IIA	2017-2020	1,136 (869-1,556)	Beckman Coulter AU480 (Beckman Coulter)	County-level scheduling variation; annual OGTT completion denominator unavailable
C24	Qinhuangdao	Urban core	Grade IIIA	2017-2023	1,617 (1,188-2,725)	Siemens ADVIA Chemistry XPT (Siemens Healthineers)	No procedural change recorded in the center summary
C25	Qinhuangdao	County/rural	Grade IIA	2017-2021	896 (661-1,275)	Beckman Coulter AU680 (Beckman Coulter)	/
C26	Zhangjiakou	Urban core	Grade IIIA	2017-2023	1,358 (1,058-2,147)	Roche cobas c702 (Roche Diagnostics)	No procedural change recorded in the center summary
C27	Zhangjiakou	County/rural	Grade IIA	2017-2020	963 (708-1,252)	Mindray BS-800M (Mindray)	County-level scheduling variation; annual OGTT completion denominator unavailable
C28	Chengde	Urban core	Grade IIIA	2017-2022	1,276 (934-2,109)	Abbott ARCHITECT c16000 (Abbott)	Temporary reduction in scheduled OGTT capacity during parts of 2022; annual completion denominator not retained
C29	Chengde	County/rural	Grade IIA	2017-2019	866 (732-1,063)	Beckman Coulter AU480 (Beckman Coulter)	County-level scheduling variation; annual OGTT completion denominator unavailable
C30	Hengshui	Urban core	Grade IIIA	2017-2023	1,252 (993-1,766)	Mindray BS-2000M (Mindray)	No procedural change recorded in the center summary
C31	Hengshui	County/rural	Grade IIA	2017-2021	864 (627-1,278)	Beckman Coulter AU680 (Beckman Coulter)	County-level scheduling variation; annual OGTT completion denominator unavailable

Note: Mean annual eligible deliveries and ranges refer to the listed participation years; means are rounded to whole deliveries and are not the hospitals' total obstetric service volumes. The annual participating-center counts reproduce Table S1C. The roster contains 11 hospitals listed throughout 2017-2023; S1C reports a 10-hospital analysis set, whose membership requires reconciliation (Q5). Absence of a procedural change in the center summary is not evidence that no unrecorded change occurred.

Supplementary Table S1B. Center-level glucose testing, sample handling, quality control, and analytic classification rule.

Center-specific screening and analytical procedures are reported below. The common analytic classification rule is defined in the table note.

Center	Method	Sample	Fasting/handling	Quality control	Screening pathway	Classification rule
C01	Hexokinase	Venous plasma; fluoride/oxalate tube	8-12 h fast; centrifuged within 30 min; analyzed within 2 h	Two-level internal QC each testing day; provincial EQA twice yearly	Universal 75-g OGTT at 24-28 weeks	Common analytic rule (see note).
C02	Hexokinase	Venous plasma; fluoride/oxalate tube	8-12 h fast; centrifuged within 30 min; analyzed within 2 h	Two-level internal QC each testing day; provincial EQA twice yearly	Universal 75-g OGTT at 24-28 weeks	Common analytic rule (see note).
C03	Hexokinase	Venous plasma; lithium-heparin tube	8-12 h fast; centrifuged within 30 min; analyzed within 2 h	Two-level internal QC each testing day; provincial EQA twice yearly	Universal 75-g OGTT at 24-28 weeks	Common analytic rule (see note).
C04	Hexokinase	Venous plasma; fluoride/oxalate tube	8-10 h fast; centrifuged within 60 min; analyzed within 2 h	Daily internal QC; provincial/inter-laboratory EQA at least yearly	75-g OGTT at 24-28 weeks; fasting-glucose triage sometimes used for scheduling	Common analytic rule (see note).
C05	Hexokinase	Venous plasma; fluoride/oxalate tube	8-10 h fast; centrifuged within 60 min; analyzed within 2 h	Daily internal QC; provincial/inter-laboratory EQA at least yearly	Universal 75-g OGTT at 24-28 weeks	Common analytic rule (see note). Reduced OGTT scheduling capacity in 2022; completion denominator unavailable.
C06	Hexokinase	Venous plasma; fluoride/oxalate tube	8-12 h fast; centrifuged within 30 min; analyzed within 2 h	Two-level internal QC each testing day; provincial EQA twice yearly	Universal 75-g OGTT at 24-28 weeks	Common analytic rule (see note).
C07	Hexokinase	Venous plasma; fluoride/oxalate tube	8-12 h fast; centrifuged within 30 min; analyzed within 2 h	Two-level internal QC each testing day; provincial EQA twice yearly	Universal 75-g OGTT at 24-28 weeks	Common analytic rule (see note).
C08	Hexokinase	Venous plasma; fluoride/oxalate tube	8-10 h fast; centrifuged within 60 min; analyzed within 2 h	Daily internal QC; provincial/inter-laboratory EQA at least yearly	75-g OGTT at 24-28 weeks; fasting-glucose triage sometimes used for scheduling	Common analytic rule (see note).
C09	Hexokinase	Venous plasma; fluoride/oxalate tube	8-12 h fast; centrifuged within 30 min; analyzed within 2 h	Two-level internal QC each testing day; provincial EQA twice yearly	Universal 75-g OGTT at 24-28 weeks	Common analytic rule (see note).
C10	Glucose oxidase/hexokinase	Venous plasma; lithium-heparin tube	8-12 h fast; centrifuged within 30 min; analyzed within 2 h	Two-level internal QC each testing day; provincial EQA twice yearly	Universal 75-g OGTT at 24-28 weeks	Common analytic rule (see note).
C11	Hexokinase	Venous plasma; fluoride/oxalate tube	8-10 h fast; centrifuged within 60 min; analyzed within 2 h	Daily internal QC; provincial/inter-laboratory EQA at least yearly	Universal 75-g OGTT at 24-28 weeks	Common analytic rule (see note).
C12	Glucose oxidase	Venous plasma; fluoride/oxalate tube	8-10 h fast; centrifuged within 60 min; analyzed within 2 h	Daily internal QC; provincial/inter-laboratory EQA at least yearly	Universal 75-g OGTT at 24-28 weeks	Common analytic rule (see note). Reduced OGTT scheduling capacity in 2022; completion denominator unavailable.
C13	Hexokinase	Venous plasma; fluoride/oxalate tube	8-12 h fast; centrifuged within 30 min; analyzed within 2 h	Two-level internal QC each testing day; provincial EQA twice yearly	Universal 75-g OGTT at 24-28 weeks	Common analytic rule (see note).
C14	Glucose oxidase	Venous plasma; fluoride/oxalate tube	8-12 h fast; centrifuged within 30 min; analyzed within 2 h	Two-level internal QC each testing day; provincial EQA twice yearly	Universal 75-g OGTT at 24-28 weeks	Common analytic rule (see note).
C15	Glucose oxidase	Venous plasma; fluoride/oxalate tube	8-10 h fast; centrifuged within 60 min; analyzed within 2 h	Daily internal QC; provincial/inter-laboratory EQA at least yearly	Universal 75-g OGTT at 24-28 weeks	Common analytic rule (see note).
C16	Hexokinase	Venous plasma; fluoride/oxalate tube	8-12 h fast; centrifuged within 30 min; analyzed within 2 h	Two-level internal QC each testing day; provincial EQA twice yearly	Universal 75-g OGTT at 24-28 weeks	Common analytic rule (see note).
C17	Hexokinase	Venous plasma; fluoride/oxalate tube	8-10 h fast; centrifuged within 60 min; analyzed within 2 h	Daily internal QC; provincial/inter-laboratory EQA at least yearly	75-g OGTT at 24-28 weeks; fasting-glucose triage sometimes used for scheduling	Common analytic rule (see note).
C18	Hexokinase	Venous plasma; lithium-heparin tube	8-12 h fast; centrifuged within 30 min; analyzed within 2 h	Two-level internal QC each testing day; provincial EQA twice yearly	Universal 75-g OGTT at 24-28 weeks	Common analytic rule (see note). Reduced OGTT scheduling capacity in 2022; completion denominator unavailable.
C19	Hexokinase	Venous plasma; fluoride/oxalate tube	8-12 h fast; centrifuged within 30 min; analyzed within 2 h	Two-level internal QC each testing day; provincial EQA twice yearly	Universal 75-g OGTT at 24-28 weeks	Common analytic rule (see note).
C20	Hexokinase	Venous plasma; fluoride/oxalate tube	8-10 h fast; centrifuged within 60 min; analyzed within 2 h	Daily internal QC; provincial/inter-laboratory EQA at least yearly	Universal 75-g OGTT at 24-28 weeks	Common analytic rule (see note).
C21	Hexokinase	Venous plasma; fluoride/oxalate tube	8-12 h fast; centrifuged within 30 min; analyzed within 2 h	Two-level internal QC each testing day; provincial EQA twice yearly	Universal 75-g OGTT at 24-28 weeks	Common analytic rule (see note).
C22	Hexokinase	Venous plasma; lithium-heparin tube	8-12 h fast; centrifuged within 30 min; analyzed within 2 h	Two-level internal QC each testing day; provincial EQA twice yearly	Universal 75-g OGTT at 24-28 weeks	Common analytic rule (see note).

Center	Method	Sample	Fasting/handling	Quality control	Screening pathway	Classification rule
C23	Hexokinase	Venous plasma; fluoride/oxalate tube	8-10 h fast; centrifuged within 60 min; analyzed within 2 h	Daily internal QC; provincial/inter-laboratory EQA at least yearly	75-g OGTT at 24-28 weeks; fasting-glucose triage sometimes used for scheduling	Common analytic rule (see note).
C24	Hexokinase	Venous plasma; fluoride/oxalate tube	8-12 h fast; centrifuged within 30 min; analyzed within 2 h	Two-level internal QC each testing day; provincial EQA twice yearly	Universal 75-g OGTT at 24-28 weeks	Common analytic rule (see note).
C25	Hexokinase	Venous plasma; fluoride/oxalate tube	8-10 h fast; centrifuged within 60 min; analyzed within 2 h	Daily internal QC; provincial/inter-laboratory EQA at least yearly	Universal 75-g OGTT at 24-28 weeks	Common analytic rule (see note).
C26	Hexokinase	Venous plasma; fluoride/oxalate tube	8-12 h fast; centrifuged within 30 min; analyzed within 2 h	Two-level internal QC each testing day; provincial EQA twice yearly	Universal 75-g OGTT at 24-28 weeks	Common analytic rule (see note).
C27	Glucose oxidase	Venous plasma; fluoride/oxalate tube	8-10 h fast; centrifuged within 60 min; analyzed within 2 h	Daily internal QC; provincial/inter-laboratory EQA at least yearly	75-g OGTT at 24-28 weeks; fasting-glucose triage sometimes used for scheduling	Common analytic rule (see note).
C28	Hexokinase	Venous plasma; lithium-heparin tube	8-12 h fast; centrifuged within 30 min; analyzed within 2 h	Two-level internal QC each testing day; provincial EQA twice yearly	Universal 75-g OGTT at 24-28 weeks	Common analytic rule (see note). Reduced OGTT scheduling capacity in 2022; completion denominator unavailable.
C29	Hexokinase	Venous plasma; fluoride/oxalate tube	8-10 h fast; centrifuged within 60 min; analyzed within 2 h	Daily internal QC; provincial/inter-laboratory EQA at least yearly	75-g OGTT at 24-28 weeks; fasting-glucose triage sometimes used for scheduling	Common analytic rule (see note).
C30	Glucose oxidase/hexokinase	Venous plasma; fluoride/oxalate tube	8-12 h fast; centrifuged within 30 min; analyzed within 2 h	Two-level internal QC each testing day; provincial EQA twice yearly	Universal 75-g OGTT at 24-28 weeks	Common analytic rule (see note).
C31	Hexokinase	Venous plasma; fluoride/oxalate tube	8-10 h fast; centrifuged within 60 min; analyzed within 2 h	Daily internal QC; provincial/inter-laboratory EQA at least yearly	75-g OGTT at 24-28 weeks; fasting-glucose triage sometimes used for scheduling	Common analytic rule (see note).

Note: Recorded pregestational and overt diabetes were excluded. Random plasma glucose alone did not define GDM. For an incomplete OGTT, an observed value meeting a GDM threshold was sufficient only after overt diabetes had been excluded; otherwise glycemic status was indeterminate and the record was excluded. Thus, incomplete non-diagnostic tests were not assigned to the unaffected group. Annual OGTT eligibility and completion denominators were unavailable. QC, quality control; EQA, external quality assessment.

Supplementary Table S1C. Annual center participation and center-composition sensitivity analysis.

Reported center-composition sensitivity analyses.

Year	Hospitals	All eligible	Stable eligible	All-center GDM-alone n (%)	Stable-center GDM-alone n (%)	Center-standardized GDM-alone % (95% CI)	All-center HDP+GDM n (%)	Stable-center HDP+GDM n (%)	Center-standardized HDP+GDM % (95% CI)
2017	28	64,629	32,037	3,084 (4.77)	1,570 (4.90)	4.92 (4.75-5.09)	566 (0.88)	282 (0.88)	0.87 (0.80-0.94)
2018	30	46,347	21,468	3,194 (6.89)	1,475 (6.87)	6.84 (6.61-7.07)	262 (0.57)	124 (0.58)	0.60 (0.53-0.67)
2019	31	46,121	20,709	4,237 (9.19)	1,881 (9.08)	9.01 (8.75-9.27)	386 (0.84)	172 (0.83)	0.82 (0.74-0.90)
2020	28	34,253	16,381	3,591 (10.48)	1,697 (10.36)	10.29 (9.97-10.61)	429 (1.25)	205 (1.25)	1.23 (1.11-1.35)
2021	24	27,645	14,952	3,652 (13.21)	1,881 (12.58)	12.52 (12.13-12.91)	383 (1.39)	208 (1.39)	1.36 (1.22-1.50)
2022	16	22,864	16,798	403 (1.76)	339 (2.02)	2.10 (1.91-2.29)	60 (0.26)	49 (0.29)	0.30 (0.23-0.37)
2023	14	22,419	18,084	439 (1.96)	380 (2.10)	2.18 (1.99-2.37)	52 (0.23)	45 (0.25)	0.27 (0.20-0.34)

Note: Stable-center estimates use the reported 10-hospital analysis set. Center-standardized estimates are marginal predictions from a binomial mixed model with a hospital random intercept and calendar-year indicators. All-center GDM denotes GDM alone, not all pregnancies with any GDM diagnosis. Annual OGTT eligibility and completion denominators were unavailable. The exact membership of the ten-hospital set, the standardization target/weights, and the CI method require documentation (Q5-Q6); the reported estimates have not been altered.

Supplementary Table S1D. Original and hospital-random-intercept outcome estimates.

Reported hospital-adjusted analyses retain the original-model covariates and add a hospital-specific random intercept.

Outcome	HDP-alone original	HDP-alone hospital-adjusted	GDM-alone original	GDM-alone hospital-adjusted	HDP+GDM original	HDP+GDM hospital-adjusted
Cesarean delivery	3.280 (3.121-3.447)	3.14 (2.95-3.35)	1.125 (1.083-1.169)	1.10 (1.05-1.15)	2.358 (2.104-2.642)	2.27 (1.99-2.59)
Placental abruption	37.441 (28.130-49.835)	34.20 (24.80-47.16)	7.672 (5.334-11.033)	7.02 (4.70-10.48)	18.898 (10.612-33.652)	17.25 (8.95-33.25)
Premature rupture of membranes	0.468 (0.416-0.525)	0.50 (0.44-0.57)	1.382 (1.303-1.466)	1.35 (1.27-1.44)	0.907 (0.747-1.102)	0.93 (0.75-1.16)
Puerperal infection	6.548 (2.842-15.085)	5.89 (2.30-15.10)	2.095 (0.712-6.162)	1.91 (0.61-5.97)	4.930 (0.660-36.816)	4.45 (0.55-36.18)
Postpartum hemorrhage	1.723 (1.506-1.972)	1.68 (1.45-1.94)	1.698 (1.519-1.898)	1.64 (1.45-1.85)	2.321 (1.779-3.028)	2.21 (1.65-2.96)
Anaemia	1.639 (1.574-1.705)	1.60 (1.53-1.68)	1.488 (1.441-1.537)	1.46 (1.40-1.51)	1.810 (1.655-1.979)	1.77 (1.61-1.95)
Hypothyroidism	1.133 (1.024-1.253)	1.11 (0.99-1.24)	1.307 (1.211-1.411)	1.28 (1.18-1.39)	1.825 (1.520-2.193)	1.77 (1.46-2.15)
Late preterm delivery	9.990 (9.193-10.857)	9.35 (8.50-10.28)	1.403 (1.304-1.510)	1.38 (1.27-1.50)	3.988 (3.481-4.568)	3.82 (3.28-4.45)
Early preterm delivery	4.786 (4.501-5.088)	4.55 (4.24-4.89)	1.540 (1.355-1.750)	1.49 (1.29-1.72)	5.543 (4.509-6.814)	5.18 (4.08-6.57)
Macrosomia	0.813 (0.752-0.879)	0.84 (0.77-0.91)	1.413 (1.344-1.458)	1.39 (1.32-1.47)	1.477 (1.288-1.694)	1.44 (1.24-1.67)
Small for gestational age	10.268 (9.592-10.991)	9.71 (8.95-10.53)	0.927 (0.809-1.062)	0.95 (0.82-1.10)	5.873 (4.967-6.944)	5.42 (4.52-6.49)
Neonatal asphyxia	3.737 (3.219-4.340)	3.54 (3.00-4.17)	1.053 (0.853-1.300)	1.02 (0.81-1.29)	2.981 (2.098-4.235)	2.78 (1.89-4.09)
Stillbirth	3.119 (1.726-5.636)	2.94 (1.55-5.58)	1.947 (1.056-3.589)	1.82 (0.94-3.51)	1.352 (0.188-9.742)	1.28 (0.17-9.60)

Note: Original-model estimates reproduce Table 4. Hospital-adjusted estimates include a hospital-specific random intercept in addition to maternal age, marital status, education, hospital level, gravidity, parity, prenatal examination frequency, and previous cesarean section. The reported median hospital ICC was 0.047 (range, 0.018-0.109). Point estimates retained their direction. The HDP-alone/hypothyroidism CI and GDM-alone/stillbirth CI included 1 after hospital adjustment. This does not establish equivalence between models or the absence of residual confounding. The macrosomia CI and HDP preterm-row mapping remain flagged for output verification (Q7).

Supplementary Table S2. Approximate E-values for positive associations with 95% CIs excluding 1.

Adjusted ORs were converted using RR ≈ OR/[(1-p0)+p0×OR], where p0 was calculated from the unrounded unaffected-group event count divided by 231,832. The E-value was RR + sqrt[RR×(RR−1)]; the same transformation was applied to the lower OR confidence limit. Displayed p0 percentages are rounded for presentation only. Because a marginal baseline proportion is combined with an adjusted OR, the results are approximate sensitivity summaries, not exact adjusted-model bounds. The calculations do not incorporate uncertainty in p0, correct residual confounding, or address selection or measurement error. All 31 positive associations whose reported CIs excluded 1 are included; this rule does not imply that inverse or non-significant associations are unimportant. Recalculate only if the source ORs, CIs, group denominator, or row mapping changes.

Exposure	Outcome	Adjusted OR (95% CI)	Unaffected risk, %	Approximate RR	E-value, point estimate	E-value, lower CI limit
HDP alone	Cesarean section	3.280 (3.121-3.447)	51.79	1.504	2.37	2.34
HDP alone	Placental abruption	37.441 (28.130-49.835)	0.04	36.924	73.35	55.18
HDP alone	Puerperal infection	6.548 (2.842-15.085)	0.01	6.544	12.57	5.13
HDP alone	Postpartum hemorrhage	1.723 (1.506-1.972)	1.30	1.707	2.81	2.36
HDP alone	Anaemia	1.639 (1.574-1.705)	31.04	1.368	2.08	2.01
HDP alone	Hypothyroidism	1.133 (1.024-1.253)	3.48	1.128	1.51	1.18
HDP alone	Late preterm delivery	9.990 (9.193-10.857)	3.56	7.566	14.61	13.71
HDP alone	Early preterm delivery	4.786 (4.501-5.088)	1.07	4.600	8.67	8.15
HDP alone	Small for gestational age	10.268 (9.592-10.991)	1.43	9.070	17.63	16.58
HDP alone	Neonatal asphyxia	3.737 (3.219-4.340)	0.54	3.683	6.83	5.82
HDP alone	Stillbirth	3.119 (1.726-5.636)	0.04	3.116	5.68	2.84
GDM alone	Cesarean section	1.125 (1.083-1.169)	51.79	1.057	1.30	1.24
GDM alone	Placental abruption	7.672 (5.334-11.033)	0.04	7.652	14.79	10.12
GDM alone	Premature rupture of membranes	1.382 (1.303-1.466)	5.71	1.352	2.04	1.88
GDM alone	Postpartum hemorrhage	1.698 (1.519-1.898)	1.30	1.683	2.75	2.39
GDM alone	Anaemia	1.488 (1.441-1.537)	31.04	1.292	1.91	1.85
GDM alone	Hypothyroidism	1.307 (1.211-1.411)	3.48	1.293	1.91	1.70
GDM alone	Late preterm delivery	1.403 (1.304-1.510)	3.56	1.383	2.11	1.90
GDM alone	Early preterm delivery	1.540 (1.355-1.750)	1.07	1.531	2.43	2.04
GDM alone	Macrosomia	1.413 (1.344-1.458)	8.43	1.365	2.07	1.94
GDM alone	Stillbirth	1.947 (1.056-3.589)	0.04	1.946	3.30	1.30
HDP+GDM	Cesarean section	2.358 (2.104-2.642)	51.79	1.384	2.11	2.01
HDP+GDM	Placental abruption	18.898 (10.612-33.652)	0.04	18.769	37.03	20.63
HDP+GDM	Postpartum hemorrhage	2.321 (1.779-3.028)	1.30	2.282	3.99	2.92
HDP+GDM	Anaemia	1.810 (1.655-1.979)	31.04	1.446	2.25	2.09
HDP+GDM	Hypothyroidism	1.825 (1.520-2.193)	3.48	1.774	2.95	2.35
HDP+GDM	Late preterm delivery	3.988 (3.481-4.568)	3.56	3.604	6.67	5.85
HDP+GDM	Early preterm delivery	5.543 (4.509-6.814)	1.07	5.287	10.05	8.16
HDP+GDM	Macrosomia	1.477 (1.288-1.694)	8.43	1.420	2.19	1.83
HDP+GDM	Small for gestational age	5.873 (4.967-6.944)	1.43	5.492	10.46	8.87
HDP+GDM	Neonatal asphyxia	2.981 (2.098-4.235)	0.54	2.950	5.35	3.59

Supplementary Table S3. Availability of HDP subtype information.

HDP subtype distributions and postpartum classification cannot be resolved from the composite results alone. No subtype-specific estimates are reported.

Requested subtype	Availability in the current report	Interpretation / unresolved definition
Gestational hypertension	Not reliably separable from the composite HDP exposure	No subtype-specific count or outcome estimate reported.
Preeclampsia and severity	Not separately quantified	Severity distribution cannot be inferred from the composite estimates.
Eclampsia	Not separately quantified	No subtype-specific estimate reported; potential sparse-event limitation.
Chronic hypertension in pregnancy	Not separately quantified	/
Chronic hypertension with superimposed preeclampsia	Not separately quantified	/
Postpartum blood-pressure recovery	Not separately quantified	/
